# The impact of bedtime alignment on sleep health in older couples: gender-sensitive analysis

**DOI:** 10.1192/bjo.2026.10982

**Published:** 2026-02-20

**Authors:** Hoyoung An, Hee Won Yang, Dae Jong Oh, Eunji Lim, Seung Wan Suh, Seonjeong Byun, Tae Hui Kim, Kyung Phil Kwak, Bong Jo Kim, Shin Gyeom Kim, Jeong Lan Kim, Seok Woo Moon, Joon Hyuk Park, Seung-Ho Ryu, Dong Woo Lee, Seok Bum Lee, Jung Jae Lee, Jin Hyeong Jhoo, Ji Won Han, Ki Woong Kim

**Affiliations:** Department of Psychiatry, Keyo Hospital, Uiwang, Republic of Korea; Department of Psychiatry, College of Medicine, Chungnam National University, Daejeon, Republic of Korea; Workplace Mental Health Institute, Kangbuk Samsung Hospital, Sungkyunkwan University School of Medicine, Seoul, Republic of Korea; Department of Psychiatry, Gyeongsang National University, School of Medicine, Jinju, Republic of Korea; Seoul Heal Mental Health Clinic, Seoul, Republic of Korea; Department of Psychiatry, Uijeongbu St Mary’s Hospital, College of Medicine, The Catholic University of Korea, Uijeongbu, Republic of Korea; Department of Psychiatry, Yonsei University Wonju Severance Christian Hospital, Wonju, Republic of Korea; Department of Psychiatry, Dongguk University Gyeongju Hospital, Gyeongju, Republic of Korea; Department of Neuropsychiatry, Soonchunhyang University Bucheon Hospital, Bucheon, Republic of Korea; Department of Psychiatry and Research Institute of Medical Science, Konkuk University, Konkuk University Chungju Hospital, Chungju, Republic of Korea; Department of Neuropsychiatry, Jeju National University Hospital, Jeju, Republic of Korea; Department of Psychiatry, School of Medicine, Konkuk University, Konkuk University Medical Center, Seoul, Republic of Korea; Department of Psychiatry, Inje University Sanggye Paik Hospital, Seoul, Republic of Korea; Department of Psychiatry, Dankook University Hospital, Cheonan, Republic of Korea; Department of Psychiatry, Kangwon National University, College of Medicine, Chuncheon, Republic of Korea; Department of Neuropsychiatry, Seoul National University Bundang Hospital, Seongnam, Republic of Korea; Department of Brain and Cognitive Science, Seoul National University College of Natural Sciences, Seoul, Republic of Korea; Department of Psychiatry, Seoul National University College of Medicine, Seoul, Republic of Korea; Institute of Human Behavioral Medicine, Seoul National University Medical Research Center, Seoul, Republic of Korea; Department of Health Science and Technology, Graduate School of Convergence Science and Technology, Seoul National University, Seoul, Republic of Korea

**Keywords:** Sleep, spouses, longitudinal studies, sex, sleep quality

## Abstract

**Background:**

Although most couples share a bed, current interventions rarely consider dyadic sleep patterns.

**Aims:**

We investigated whether bedtime alignment between partners affects longitudinal sleep outcomes in older couples, with particular attention to gender differences.

**Method:**

Based on the temporal relationship between partners’ bedtimes and the earlier sleeper’s sleep onset latency, 859 couples (1718 individuals) aged ≥60 years were classified into 5 mutually exclusive bedtime alignment groups. Pittsburgh Sleep Quality Index (PSQI) scores, sleep onset latency and sleep efficiency were compared using analysis of variance and multivariate analysis of covariance. Both cross-sectional and 8-year longitudinal trajectory analyses were conducted.

**Results:**

Bedtime alignment significantly affected sleep outcomes (*P* < 0.001, Pillai’s Trace = 0.37, *F*_24_, _3352_ = 14.04, *P* < 0.001, *η* ^2^ *P* = 0.09). Couples with synchronised bedtimes demonstrated excellent sleep quality, whereas those with bedtime differences less than the earlier sleeper’s sleep onset latency exhibited the worst. The earlier sleepers in such couples experienced longer sleep onset latencies (53.4 ± 46.8 min) and greater sleep quality impairment (PSQI = 7.9 ± 4.1). The 8-year trajectory analysis revealed gender-specific vulnerability: only women in misaligned groups experienced progressive sleep deterioration over time (5.84 ± 8.42 min/year increase in sleep onset latency, *P* < 0.001; 1.27 ± 1.93%/year decrease in sleep efficiency, *P* < 0.001), whereas men maintained stable sleep parameters regardless of alignment.

**Conclusions:**

Bedtime alignment represents a modifiable determinant of sleep health in older couples, with synchronised bedtimes providing optimal outcomes and partial sleep onset overlap creating disruption. This particularly benefits women, who show progressive deterioration with misalignment. These findings support the development of gender-informed, couple-based interventions for sleep disorders.

Sleep disturbances affect 40–46% of older adults globally^1^ and increase all-cause mortality by 14–34%, cardiovascular disease by 12–46% and dementia risk by 19–51%.^2–4^ However, current individual-focused interventions, such as cognitive–behavioural treatment for insomnia (CBT-I), a short, structured treatment technique that aims to restructure thoughts and behaviours related to sleep, show limited effectiveness, necessitating novel therapeutic approaches. Although 89% of couples share a bed^5^ and demonstrate 55–88% bedtime alignment (i.e. synchronised bedtimes),^6^ clinical sleep assessments rarely consider dyadic patterns.^7^

Recent evidence indicates that bedtime alignment is crucial for sleep outcomes of couples and, being a modifiable social determinant,^8^ it may represent a promising intervention target for older couples. Preliminary evidence suggests that synchronised bedtimes may enhance sleep outcomes through reduced sleep disruptions from partner movements, reinforced circadian entrainment and psychological benefits of shared routines.^9^ Conversely, misaligned bedtimes may fragment sleep architecture and compromise restorative functions of sleep, potentially amplifying age-related vulnerabilities.^7,9^ An analysis of 47 420 couples from the UK Biobank further substantiated these findings, demonstrating bidirectional influences and cross-trait associations among sleep duration, sleep efficiency, nocturnal sleep episodes and bedtime alignment, suggesting that bedtime alignment may be linked to other sleep outcomes within couples.^10^ However, these studies could not define optimal timing strategies for bed-sharing couples, and their cross-sectional designs precluded any inferences regarding causality. Gender adds crucial complexity: women report 1.4 times higher insomnia rates than men,^11^ with post-menopausal women showing the greatest vulnerability due to hormonal changes disrupting circadian rhythms and increasing sleep fragmentation.^12,13^ These gender-specific vulnerabilities suggest that bedtime alignment interventions may differentially benefit women and men, but longitudinal gender-specific effects remain unexplored. This study addresses these gaps by investigating bedtime alignment patterns and sleep outcomes in 859 older couples over an 8-year period. We hypothesised that (a) misaligned bedtimes within couples would show worse sleep outcomes than synchronised bedtimes; and (b) the effects would differ significantly by gender, with women demonstrating greater vulnerability to misalignment-related sleep deterioration over time.

## Method

### Study design and participants

**Fig. 1:**
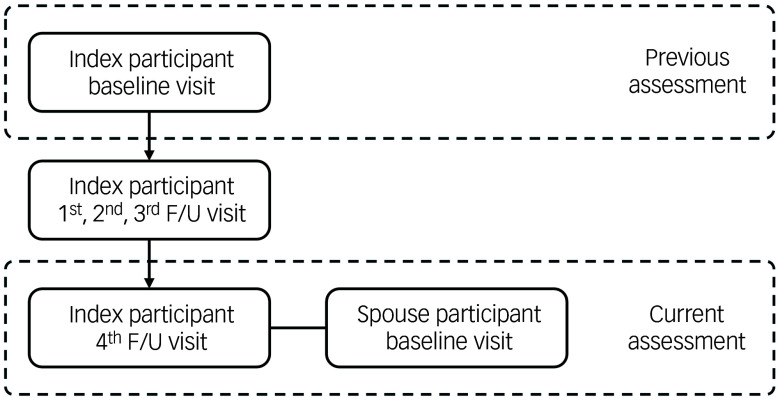
Assessments of the study subjects. F/U, follow-up.

This cross-sectional and longitudinal trajectory dyadic analysis utilised data from the Korean Longitudinal Study on Cognitive Aging and Dementia (KLOSCAD), a nationwide prospective cohort study investigating cognitive decline and dementia among Korean adults aged 60 years and older.^14^ Participants in KLOSCAD were recruited through stratified random sampling from residential registers across 13 districts in South Korea, and followed biennially (hereafter, ‘index participants’). The initial assessment took place between November 2010 and October 2012, and the fourth follow-up was conducted between November 2018 and October 2020. At the fourth follow-up assessment, spouses of the index participants were invited to participate together to construct a couple cohort, and 865 spouses responded (hereafter ‘spouse participants’) (Fig. 1). Therefore, whereas index participants had five assessments, spouse participants had only one, and thus longitudinal trajectory analysis included only the former. After excluding 6 couples with missing key variables, 859 couples (1718 individuals) were included in the final analytic sample. All couples were heterosexual, married and cohabiting at the fourth follow-up assessment. The study design allows for actor–partner interdependence model analysis to examine both individual (actor) and cross-partner (partner) effects within couples.

### Ethical standards

The authors assert that all procedures contributing to this work comply with the ethical standards of the relevant national and institutional committees on human experimentation, and with the Helsinki Declaration of 1975 as revised in 2013. All procedures involving human subjects/patients were approved by the Institutional Review Board of Seoul National University Bundang Hospital (IRB no. B-0912-089-010). Written informed consent was obtained from all participants or their legally authorised representatives. This study also follows the Strengthening the Reporting of Observational Studies in Epidemiology guidelines for cross-sectional studies.

### Assessment of sleep outcomes

Sleep quality was evaluated using the Pittsburgh Sleep Quality Index (PSQI), a validated, 19-item, self-report instrument assessing 7 domains, including sleep quality, latency, duration, efficiency, disturbance, medication use and daytime dysfunction. Higher PSQI scores indicate poorer subjective sleep quality.^15^ Sleep onset latency, defined as the time required to fall asleep after retiring to bed, was self-reported in minutes for the previous month. Although sleep onset latency is incorporated into the PSQI total score as an ordinal item, its raw value in minutes was included in all analyses in which it was examined independently. Sleep efficiency was calculated as the ratio of total sleep time to time in bed, expressed as a percentage.^16^ Although PSQI contains an item assessing sleep efficiency, this component is not incorporated into the total score and was treated as a continuous variable in all analyses.

### Classification of bedtime alignment

Couples were classified into five mutually exclusive bedtime alignment groups based on the temporal relationship between partners’ bedtimes and sleep onset latencies, representing a novel approach to quantifying couple sleep synchrony. The first group (index much earlier, ‘I >> S’) comprised couples in which the index participant retired to bed earlier than their spouse, with the difference in bedtimes exceeding the index participant’s sleep onset latency. The second group (index earlier, ‘I > S’) comprised couples where the index participant retired to bed earlier than their spouse, but the difference in bedtimes was shorter than the index participant’s sleep onset latency. The third group, (synchronised, ‘I = S’) consisted of couples who retired to bed at the same time. The fourth group (index later, ‘I < S’) comprised couples where the index participant retired to bed later than their spouse, with the difference in bedtimes being shorter than the spouse participant’s sleep onset latency. The final group (index much later, ‘I << S’) comprised couples where the index participant retired to bed later than their spouse, and the difference in bedtimes exceeded the spouse participant’s sleep onset latency. Details of the calculations are provided in Supplementary Table 1 available at https://doi.org/10.1192/bjo.2026.10982. This classification system, which was based on specific questions targeting bedtimes and sleep onset latency, considers both objective bedtime differences and individual sleep onset characteristics, providing a clinically meaningful framework for understanding a couple’s sleep dynamics. It was created by the authors for this study, and therefore has not yet been independently validated.

### Assessment of covariates

Covariates were selected based on established associations with sleep outcomes in older adults and included demographic, physical and psychosocial factors.^17^ Demographic variables included age, gender and education level. Physical activity was quantified as metabolic equivalent task.^18^ Depressive symptoms were evaluated using the 15-item Geriatric Depression Scale (GDS).^19^ Comorbidities were assessed using the Cumulative Illness Rating Scale (CIRS), which evaluates disease burden across 13 organ systems.^20^ These variables were selected based on their established associations with sleep outcomes.^17^ Sleep-related covariates included rapid-eye movement (REM) sleep behaviour disorder (assessed using the REM sleep Behavior Disorder Screening Questionnaire)^21^ and sleep apneoa (evaluated by the STOP questionnaire for obstructive sleep apnoea).^22^

### Statistical analysis

Demographic and clinical characteristics were summarised using means (standard deviations) for continuous variables, and frequencies (percentages) for categorical variables. Between-group differences in bedtime alignment categories were analysed using one-way analysis of variance (ANOVA) for continuous variables and chi-square tests for categorical variables. Differences between the index and spouse participants within each bedtime group were examined using Student’s *t*-tests and chi-square tests.

Differences in sleep outcomes across bedtime alignment groups were examined using ANOVA, with separate analyses for PSQI, sleep onset latency and sleep efficiency. Additionally, due to the intercorrelated nature of sleep outcomes (PSQI, sleep onset latency and sleep efficiency), multivariate analysis of covariance (MANCOVA) was employed to simultaneously analyse these variables while controlling for their interdependence and reducing type I error risk compared with multiple univariate tests, with adjustments for covariates that differed significantly between groups at *α* = 0.05. Within-couple comparisons (index versus spouse participants) were conducted using Student’s *t*-tests and chi-square tests, with additional adjustment for demographic and clinical variables that differed between partners.

Because spouse participants had only a single assessment, longitudinal trajectory analyses were conducted for index participants only. We examined average annual changes in sleep outcomes over the 8-year follow-up period across bedtime alignment groups using ANOVA, with separate analyses for PSQI, sleep onset latency and sleep efficiency and MANCOVA. Because sleep outcomes show interdependent changes over time, MANCOVA allowed us to capture the overall trajectory of their deterioration or improvement while accounting for correlations among PSQI scores, sleep onset latency and sleep efficiency changes. Because bedtime alignment was assessed only at the fourth follow-up (not at baseline), participants were classified by their current alignment pattern. This approach enabled examination of how different developmental trajectories – culminating in specific alignment patterns – relate to corresponding sleep outcome trajectories. The analysis addressed whether individuals who eventually developed misaligned bedtimes showed different deterioration patterns in sleep outcomes compared with those who maintained synchronisation. Adjustments included initial values of the outcome variable and initial GDS and CIRS scores. MANCOVA models additionally adjusted for participant age, inter-partner age difference, index participant gender, earlier partner’s bedtime and current assessment GDS and CIRS scores for both partners.

To control type I error inflation, Bonferroni corrections were applied for all *post hoc* pairwise comparisons. Statistical significance was set at *P* < 0.05 for primary analyses. All analyses were performed using R version 4.1.2 for Windows (R Core Team, Vienna, Austria; https://cran.r-project.org) with the following packages: car (ANCOVA), chisq.posthoc.test (Bonferroni *post hoc* tests for chi-square tests), dplyr (data manipulation), emmeans (Bonferroni *post hoc* tests for ANOVA), ggplot2 (data visualisation) and jmv (MANCOVA).

## Results

### Characteristics of the study population

The bedtime alignment distribution revealed distinct patterns: 274 couples (31.9%) were classified as I >> S (index much earlier), 48 (5.6%) as I > S (index earlier), 201 (23.4%) as I = S (synchronised), 55 (6.4%) as I < S (index later) and 281 (32.7%) as I << S (index much later). This distribution demonstrates that nearly two-thirds of older couples (64.6%) maintained significant bedtime misalignment, with synchronised bedtimes observed in less than a quarter of couples. Gender distribution differed significantly across bedtime alignment groups (*P* < 0.001), with women comprising 50.9% of index participants in the I << S group compared with only 28.8% in the I >> S group. Index participants in the I > S group exhibited the highest burden of depressive symptoms (GDS = 11.5 ± 7.4) and comorbidities (CIRS = 8.2 ± 4.1). Similarly, spouse participants in the I < S group demonstrated elevated depressive symptoms (GDS = 10.8 ± 6.8), suggesting that partial sleep onset overlap may reflect underlying health vulnerabilities (Supplementary Table 2).

### Bedtime alignment and cross-sectional sleep outcomes

The most striking finding was that partial sleep onset overlap groups – groups where one partner was joined in bed before they fell asleep – demonstrated markedly worse sleep outcomes compared with both synchronised and completely separated couples across all parameters (Pillai’s Trace = 0.37, *F*_24_, _3352_ = 14.04, *P* < 0.001, *η* ^2^ *P* = 0.09, medium to large effect; Supplementary Table 2).

**Fig. 2:**
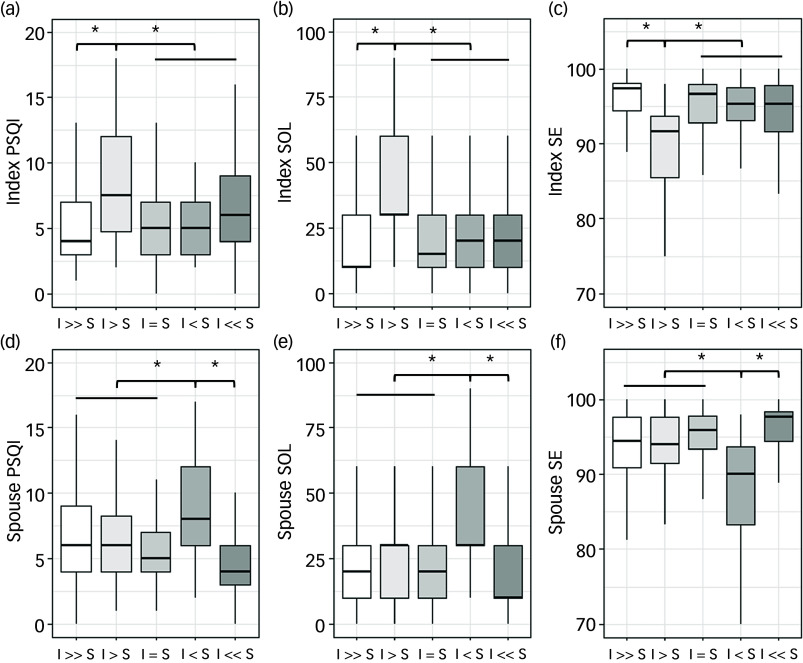
Comparison of sleep parameters between bedtime groups for index and spouse participants. (a), (b), (c) Index participants. (d), (e), (f) Spouse participants. Couples were classified into five mutually exclusive bedtime alignment groups based on the temporal relationship between partners’ bedtimes and sleep onset latencies: (1) I >> S (index much earlier) group comprised couples in which the index participant retired to bed earlier than their spouse, with the difference in bedtimes exceeding the index participant’s sleep onset latency; (2) I > S (index earlier) group comprised couples where the index participant retired to bed earlier than their spouse, but the difference in bedtimes was shorter than the index participant’s sleep onset latency; (3) I = S (synchronised) group consisted of couples who retired to bed at the same time; (4) I < S (index later) group comprised couples where the index participant retired to bed later than their spouse, with the difference in bedtimes shorter than the spouse participant’s sleep onset latency; and (5) I << S (index much later) group comprised couples where the index participant retired to bed later than their spouse, and the difference in bedtimes exceeded the spouse participant’s sleep onset latency. PSQI, Pittsburgh Sleep Quality Index (point); SOL, sleep onset latency (min); SE, sleep efficiency (%). *P < 0.05 by one-way analysis of variance with Bonferroni *post hoc* comparisons.

Participants experiencing partial overlap – those who go to bed earlier than their partner but are still awake before their partner joins the bed – showed substantial differences in all sleep outcomes. Index participants in the I > S group had PSQI scores of 7.9 ± 4.1 *v*. 5.9 ± 3.4 for synchronised couples (*P* < 0.001 for overall group differences, Cohen’s *d* = 0.40 for between-group comparison), and a sleep onset latency of 53.4 ± 46.8 *v*. 22.4 ± 21.1 min (*P* < 0.001, Cohen’s *d* = 1.21). Similarly, spouse participants in the I < S group showed a sleep onset latency of 63.2 ± 49.7 *v*. 24.0 ± 21.0 min for synchronised couples (*P* < 0.001, Cohen’s *d* = −1.29), with sleep efficiency of 85.6 ± 12.6 *v*. 94.3 ± 5.7% (*P* < 0.001, Cohen’s *d* = 1.19). Couples in the I = S group consistently demonstrated favourable sleep outcomes across all parameters, with both partners achieving sleep onset latencies, sleep efficiency and overall sleep quality scores that were among the best observed across all bedtime alignment categories. Couples with complete separation (I >> S and I << S) showed intermediate values – better than partial overlap groups but not as favourable as synchronised couples (Fig. 2 and Supplementary Table 2).

Gender-stratified analyses revealed that bedtime alignment effects were significant in both men and women, although with different patterns (Tables 1 and 2). Bedtime alignment remained significantly associated with sleep outcomes in women (adjusted *F*_24_, _1330_ = 7.34, *P* < 0.001) and men (adjusted *F*_24_, _1972_ = 7.48, *P* < 0.001). However, women consistently demonstrated poorer sleep across all groups among index participants (Table 2): higher PSQI scores (6.4 ± 3.5 *v*. 5.6 ± 3.2 in men, *P* = 0.002), longer sleep onset latency (26.4 ± 27.4 *v*. 21.9 ± 22.3 min, *P* = 0.012) and lower sleep efficiency (93.5 ± 8.3 *v*. 94.9 ± 5.4%, *P* = 0.006). This gender disparity was even greater among spouse participants, with women showing markedly worse sleep outcomes: PSQI scores of 6.6 ± 3.7 *v*. 5.1 ± 3.1 in men (*P* < 0.001), sleep onset latency of 30.6 ± 33.2 *v*. 20.3 ± 23.5 min (*P* < 0.001) and sleep efficiency of 92.7 ± 7.9 *v*. 95.3 ± 5.7% (*P* < 0.001).

**Table 1:** Effects of bedtime group on current sleep parameters

Variables	Index						Spouse					
	PSQI		SOL		SE		PSQI		SOL		SE	
	*F*	*P*	*F*	*P*	*F*	*P*	*F*	*P*	*F*	*P*	*F*	*P*
All (*N* = 859)												
Unadjusted^a^	11.1	<0.001	24.4	<0.001	23.9	<0.001	21.7	<0.001	35.6	<0.001	35.5	<0.001
Adjusted^b^	13.6	<0.001	26.6	<0.001	21.1	<0.001	27.1	<0.001	39.6	<0.001	39.9	<0.001
Men (*n* = 510)^c^												
Unadjusted^a^	5.7	<0.001	8.9	<0.001	10.4	<0.001	14.6	<0.001	19.2	<0.001	19.9	<0.001
Adjusted^d^	6.8	<0.001	9.6	<0.001	11.3	<0.001	18.9	<0.001	21.4	<0.001	22.3	<0.001
Women (*n* = 349)^c^												
Unadjusted^a^	4.1	0.003	16.0	<0.001	13.5	<0.001	5.3	<0.001	16.5	<0.001	14.4	<0.001
Adjusted^d^	5.0	<0.001	18.7	<0.001	15.8	<0.001	5.9	<0.001	17.0	<0.001	14.9	<0.001

PSQI, Pittsburgh Sleep Quality Index; SOL, sleep onset latency; SE, sleep efficiency.

a. Multivariate analysis of variance, with the bedtime group as the independent variable and the PSQI score, SOL and SE of the index and spouse participants as the dependent variables (Pillai’s Trace = 0.34, *F*_24, 3404_ = 13.31, *P* < 0.001 for all couples; Pillai’s Trace = 0.30 *F*_24, 2012_ = 6.71, *P* < 0.001 for couples in which the index participants were men; Pillai’s Trace = 0.43, *F*_24, 1364_ = 6.93, *P* < 0.001 for couples in which the index participants were women).

b. Adjusted for the age of the index participant, age difference between the index and spouse participants, gender of the index participant, bedtime of the participant in each couple who went to bed earlier, Geriatric Depression Scale and Cumulative Illness Rating Scale scores of the index and spouse participants and rapid eye movement (REM) sleep Behavior Disorder Screening Questionnaire score of the spouse participant (Pillai’s Trace = 0.37, *F*_24, 3352_ = 14.04, *P* < 0.001 for all couples).

c. Gender of index participants.

d. Adjusted for the age of the index participant, age difference between the index and spouse participants, bedtime of the participant in each couple who went to bed earlier, Geriatric Depression Scale and Cumulative Illness Rating Scale scores of the index and spouse participants and REM sleep Behavior Disorder Screening Questionnaire score of the spouse participant (Pillai’s Trace = 0.33, *F*_24, 1972_ = 7.48, *P* < 0.001 for couples in which the index participants were men; Pillai’s Trace = 0.47, *F*_24, 1330_ = 7.34, *P* < 0.001 for the couples in which the index participants were women).

**Table 2:** Comparison of demographic, clinical and sleep characteristics of participants by gender

Variables	Previous assessment^a^			Current assessment					
	Index			Index			Spouse		
	Men	Women	*P* ^b^	Men	Women	*P* ^b^	Men	Women	*P* ^b^
Age, years	67.2 (5.0)	66.2 (4.5)	0.001	75.3 (5.0)	74.3 (4.5)	0.002	77.5 (4.9)	71.1 (5.4)	<0.001
Education, years	11.4 (4.7)	8.5 (4.9)	<0.001	11.4 (4.7)	8.5 (4.9)	<0.001	11.8 (6.9)	8.9 (5.3)	<0.001
Physical activity^c^	2503 (3643)	1468 (2493)	<0.001	1531 (2030)	1250 (2114)	0.053	1706 (2239)	1004 (1337)	<0.001
Current drinking^d^	105 (20.6)	0 (0)	<0.001	38 (7.5)	0 (0)	<0.001	31 (8.9)	1.0 (0.2)	<0.001
GDS, points	8.0 (5.9)	9.4 (6.2)	0.001	7.7 (6.2)	8.7 (6.2)	0.016	7.5 (6.0)	9.1 (6.4)	<0.001
CIRS, points	4.6 (2.9)	4.1 (2.7)	0.006	7.3 (3.5)	5.9 (2.9)	<0.001	6.3 (3.3)	5.3 (3.1)	<0.001
RBDSQ, points	1.5 (1.8)	1.8 (2.0)	0.060	1.5 (2.0)	1.2 (1.6)	0.020	1.1 (1.6)	1.4 (1.7)	0.006
STOP, points	1.2 (1.1)	1.1 (0.9)	0.058	1.0 (0.9)	0.9 (0.7)	0.033	0.9 (0.9)	0.9 (0.8)	0.627
Bedtime, hours:minutes	22:42 (01:24)	22:00 (01:14)	<0.001	22:18 (01:14)	22:18 (01:13)	0.746	22:00 (01:14)	22:36 (01:22)	<0.001
PSQI, points	5.4 (2.9)	5.1 (3.1)	0.155	5.6 (3.2)	6.4 (3.5)	0.002	5.1 (3.1)	6.6 (3.7)	<0.001
SOL, min	20.6 (19.1)	20.3 (23.5)	0.837	21.9 (22.3)	26.4 (27.4)	0.012	20.3 (23.5)	30.6 (33.2)	<0.001
SE, %	95.0 (4.9)	95.3 (5.7)	0.374	94.9 (5.4)	93.5 (8.3)	0.006	95.3 (5.7)	92.7 (7.9)	<0.001

GDS, Geriatric Depression Scale; CIRS, Cumulative Illness Rating Scale; RBDSQ, rapid eye movement (REM) sleep Behavior Disorder Screening Questionnaire; STOP, the STOP questionnaire for obstructive sleep apnoea; PSQI, Pittsburgh Sleep Quality Index; SOL, sleep onset latency; SE, sleep efficiency.

Of the 859 participants in the index group, 510 were males, with 510 women in the spouse group.

Mean (standard deviation) for continuous variables and as number (percentage) for categorical variables.

a. The baseline assessment of the Korean Longitudinal Study on Cognitive Aging and Dementia, which had been conducted 8 years prior to the current assessment.

b. Student’s *t*-test for continuous variables and chi-square test for categorical variables.

c. Metabolic equivalent task **×** min/week.

d. ≥ 21 standard units/week.

### Sleep timing and sleep quality trajectories by current bedtime alignment pattern

Eight-year trajectory analysis revealed progressive polarisation of sleep timing. Participants currently in the I >> S group had consistently advanced their bedtimes (−6.63 ± 8.25 min/year, *P* < 0.001), whereas those in I << S group showed progressive delays (+6.10 ± 9.83 min/year, *P* < 0.001) (Table 3).

**Table 3:** Annual changes in sleep parameters of index participants over 8 years

Variables	All	Bedtime group^a^						
		I >> S	I > S	I = S	I < S	I << S	*P* ^b^	*Post-hoc* ^b^
Bedtime, min/year								
All (*N* = 859)	−0.72 (9.70)	−6.63 (8.25)	−3.17 (5.77)	−2.14 (6.23)	0.72 (6.46)	6.10 (9.83)	<0.001	a, b, c, d < e
Men (*n* = 510)	−2.82 (8.40)	−4.89 (8.36)	−1.58 (7.16)	−3.74 (7.87)	−1.44 (6.54)	0.26 (8.63)	<0.001	a, c < e
Women (*n* = 349)	2.26 (10.60)	−10.80 (6.29)	−4.76 (3.40)	0 (0)	4.82 (3.86)	11.62 (7.43)	<0.001	a < b < c < d < e
*P* ^c^	<0.001	<0.001	0.089	<0.001	0.012	<0.001		
PSQI, points/year								
All (*N* = 859)	0.08 (0.46)	−0.01 (0.39)	0.19 (0.59)	0.08 (0.45)	−0.07 (0.38)	0.16 (0.48)	<0.001	a, d < b, e
Men (*n* = 510)	0.02 (0.38)	−0.02 (0.36)	0.10 (0.45)	0.03 (0.34)	−0.00 (0.33)	0.07 (0.44)	0.010	
Women (*n* = 349)	0.16 (0.53)	0.02 (0.46)	0.29 (0.71)	0.15 (0.57)	−0.20 (0.44)	0.26 (0.50)	<0.001	d < b, e
*P* ^c^	<0.001	0.310	0.719	0.195	0.855	0.115		
SOL, min/year								
All (*N* = 859)	0.41 (3.46)	−0.23 (2.57)	3.56 (6.68)	0.36 (2.77)	−0.89 (3.85)	0.78 (3.43)	<0.001	a, c, d, e < b
Men (*n* = 510)	0.17 (2.76)	−0.10 (2.28)	1.46 (3.59)	0.20 (2.51)	0.30 (1.35)	0.28 (3.54)	0.001	
Women (*n* = 349)	0.75 (4.25)	−0.55 (3.17)	5.84 (8.42)	0.58 (3.10)	−3.15 (5.71)	1.26 (3.25)	<0.001	a, d < e < b
*P* ^c^	0.047	0.943	0.381	0.405	0.479	0.788		
SE, %/year								
All (*N* = 859)	0.08 (1.80)	0.42 (2.07)	−0.46 (2.45)	0.07 (1.37)	0.18 (0.93)	−0.16 (1.74)	<0.001	b, e < a
Men (*n* = 510)	0.30 (2.09)	0.51 (2.40)	0.28 (2.67)	0.20 (1.68)	−0.07 (0.31)	0.18 (2.08)	<0.001	
Women (*n* = 349)	−0.23 (1.21)	0.20 (0.76)	−1.27 (1.93)	−0.12 (0.73)	0.66 (1.44)	−0.47 (1.30)	<0.001	b, e < a, d
*P* ^c^	0.018	0.798	0.494	0.335	0.718	0.417		

PSQI, Pittsburgh Sleep Quality Index; SOL, sleep onset latency; SE, sleep efficiency.

Data presented as mean (standard deviation).

a. Couples were classified into five mutually exclusive bedtime alignment groups based on the temporal relationship between partners’ bedtimes and sleep onset latencies: I >> S group, in which the index participant retired to bed earlier than their spouse, with the difference in bedtimes exceeding the index participant’s sleep onset latency); I > S group, where the index participant retired to bed earlier than their spouse, but the difference in bedtimes was shorter than the index participant’s sleep onset latency; I = S group consisted of couples who retired to bed at the same time; I < S group, where the index participant retired to bed later than their spouse, with the difference in bedtimes shorter than the spouse participant’s sleep onset latency; and I << S group, where the index participant retired to bed later than their spouse, and the difference in bedtimes exceeded the spouse participant’s sleep onset latency.

b. Comparisons between bedtime groups using analysis of variance with Bonferroni *post hoc* comparisons, adjusted for gender, initial Geriatric Depression Scale score and initial Cumulative Illness Rating Scale score; and the values of the dependent sleep measure at the previous assessment, which was conducted 8 years before the current assessment. The letters a, b, c, d and e correspond to the I >> S, I > S, I = S, I < S and I << S groups, respectively.

c. Comparisons between genders using analysis of variance with Bonferroni *post hoc* comparisons, adjusted for age, years of education, physical activity, current drinking, Geriatric Depression Scale score and Cumulative Illness Rating Scale score.

**Fig. 3:**
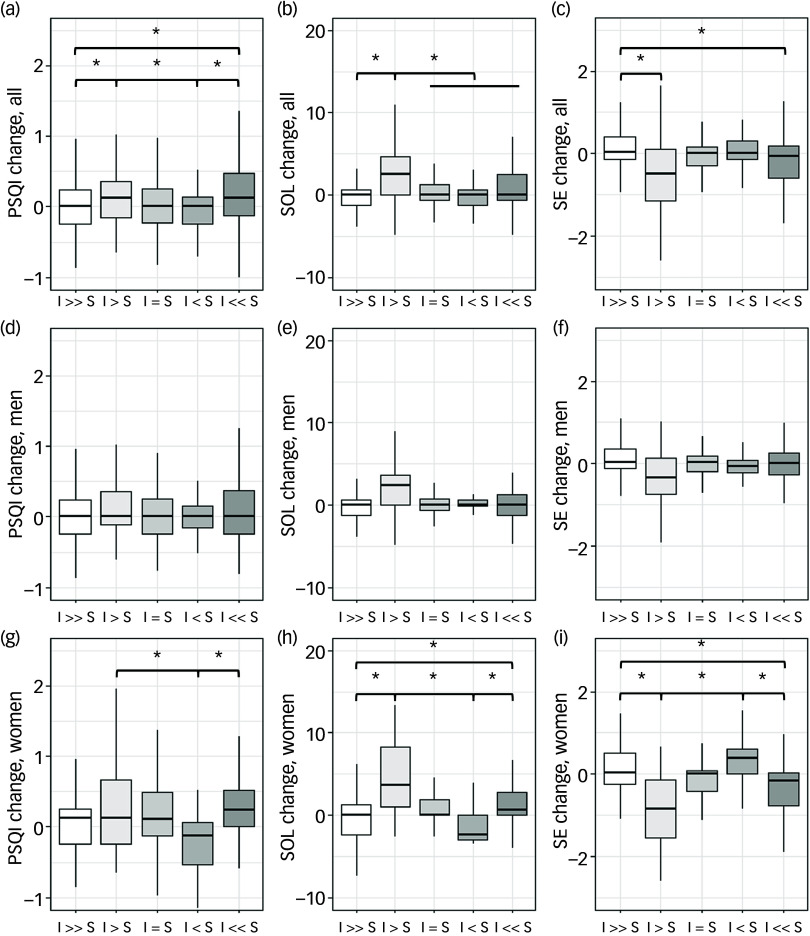
Average annual changes in sleep parameters among index participants over 8 years. (a), (b), (c) All index participants. (d), (e), (f) Men. (g), (h), (i) Women. Couples were classified into five mutually exclusive bedtime alignment groups based on the temporal relationship between partners’ bedtimes and sleep onset latencies: (1) I >> S (index much earlier) group comprised couples in which the index participant retired to bed earlier than their spouse, with the difference in bedtimes exceeding the index participant’s sleep onset latency; (2) I > S (index earlier) group comprised couples where the index participant retired to bed earlier than their spouse, but the difference in bedtimes was shorter than the index participant’s sleep onset latency; (3) I = S (synchronised) group consisted of couples who retired to bed at the same time; (4) I < S (index later) group comprised couples where the index participant retired to bed later than their spouse, with the difference in bedtimes shorter than the spouse participant’s sleep onset latency; and (5) I << S (index much later) group comprised couples where the index participant retired to bed later than their spouse, and the difference in bedtimes exceeded the spouse participant’s sleep onset latency. PSQI, Pittsburgh Sleep Quality Index (point); SOL, sleep onset latency (min); SE, sleep efficiency (%). *P < 0.05 by one-way analysis of variance with Bonferroni *post hoc* comparisons.

Gender-specific trajectory analysis revealed striking differences in long-term sleep outcomes. Among index participants, bedtime alignment groups showed significant differences in annual sleep outcome changes (Pillai’s Trace = 0.10, *F*_12_, _2535_ = 7.28, *P* < 0.001), but this was driven entirely by women (Pillai’s Trace = 0.25, *F*_12_, _1008_ = 7.75, *P* < 0.001), whereas men showed no significant group differences (Pillai’s Trace = 0.03, *F*_12_, _1494_ = 1.16, *P* = 0.306) (Table 4). Women currently in the I > S bedtime alignment group experienced the most rapid deterioration in sleep outcomes over the 8-year period (Table 3 and Fig. 3): annual increases in sleep onset latency of 5.84 ± 8.42 min/year (*P* < 0.001) and annual decreases in sleep efficiency of 1.27 ± 1.93% per year (*P* < 0.001). These rates of decline were substantially greater than those observed in women currently in synchronised trajectory couples (I = S group, 0.58 ± 3.10 min/year increase in sleep onset latency, Cohen’s *d* = 1.56 for between-group comparison; 0.12 ± 0.73% per year decrease in sleep efficiency, Cohen’s *d* = –1.07 for between-group comparison). Notably, men showed no significant differences in sleep trajectory patterns across bedtime alignment groups, suggesting that the long-term impact of bedtime alignment patterns may be gender-specific, with women demonstrating greater vulnerability to trajectory-related sleep outcome changes.

**Table 4:** Effects of bedtime groups on annual changes in sleep parameters of index participants over 8 years

Variables	PSQI		SOL		SE	
	*F*	*P*	*F*	*P*	*F*	*P*
All (*N* = 859)						
Unadjusted^a^	7.3	<0.001	16.1	<0.001	4.8	<0.001
Adjusted^b^	7.7	<0.001	16.9	<0.001	4.9	<0.001
Men (*n* = 510)						
Unadjusted^a^	1.3	0.263	1.9	0.105	0.9	0.439
Adjusted^c^	1.3	0.261	2.0	0.100	1.0	0.433
Women (*n* = 349)						
Unadjusted^a^	5.3	<0.001	17.3	<0.001	12.2	<0.001
Adjusted^c^	5.9	<0.001	19.2	<0.001	13.3	<0.001

PSQI, Pittsburgh sleep quality index; SOL, sleep onset latency; SE, sleep efficiency.

a. Multivariate analysis of variance, with bedtime group as the independent variable, and annual changes in PSQI score, SOL and SE of index participants over the past 8 years as dependent variables (Pillai’s Trace = 0.10, *F*_12, 2562_ = 7.00, *P* < 0.001 for all couples; Pillai’s Trace = 0.03 *F*_12, 1515_ = 1.14, *P* = 0.319 for couples in which the index participants were men; Pillai’s Trace = 0.24, *F*_12, 1032_ = 7.35, *P* < 0.001 for couples in which the index participants were women).

b. Adjusted for the age of the index participant, age difference between the index and spouse participants, gender of the index participant, bedtime of the participant in each couple who went to bed earlier, Geriatric Depression Scale and Cumulative Illness Rating Scale scores of the index and spouse participants (Pillai’s Trace = 0.10, *F*_12, 2535_ = 7.28, *P* < 0.001 for all couples).

c. Adjusted for the age of the index participant, age difference between the index and spouse participants, bedtime of the participant in each couple who went to bed earlier, Geriatric Depression Scale and Cumulative Illness Rating Scale scores of the index and spouse participants (Pillai’s Trace = 0.03 *F*_12, 1494_ = 1.16, *P* = 0.306 for couples in which the index participants were men; Pillai’s Trace = 0.25, *F*_12, 1008_ = 7.75, *P* < 0.001 for couples in which the index participants were women).

## Discussion

The present study established a significant association between bedtime alignment and sleep quality, sleep onset latency and sleep efficiency in older couples. Synchronised bedtimes (I = S) exhibited comparatively improved sleep, whereas partial sleep onset overlap (I > S or I < S) engendered adverse effects on all three sleep outcomes, particularly in women. The findings underscore the significance of bedtime alignment as a modifiable factor, with considerable ramifications for both public health and clinical practice.

### Temporal dimension of bedtime alignment

This study makes a significant contribution to the extant literature on couple-level sleep dynamics by placing emphasis on the temporal dimension of bedtime alignment as a critical determinant of sleep outcomes.

Previous research has demonstrated that minute-by-minute concordance in couples’ sleep–wake patterns ranges from 53 to 88%, substantially higher than for randomly matched pairs, indicating natural synchronisation processes in intimate relationships.^6^ However, few studies have explored the long-term effects of specific bedtime timing patterns or developed clinically relevant classification systems. Our novel, five-category classification system extends beyond the binary synchronised/non-synchronised approaches used in previous research, revealing that the temporal relationship between bedtime discrepancy and sleep onset latency determines sleep outcomes. When bedtime discrepancy between partners is less than the sleep onset latency of the earlier sleeper (partial overlap), that partner experiences reduced sleep quality, increased sleep onset latency and decreased sleep efficiency. This finding aligns with previous work by Hasler and Troxel, who found that discordant bedtimes predict negative partner interactions the following day, but extends this understanding by quantifying specific timing thresholds.^23^

One plausible explanation for the detrimental effects of partial overlap is that when one partner retires to bed earlier, they are more susceptible to disruptive stimuli, such as their partner entering the shared sleeping environment, during the vulnerable initial stages of sleep.^7,24^ After these stages have passed, such susceptibility probably decreases, and being joined in bed becomes less of a disruption. Beyond the physiological disruptions, bedtime alignment may also impact emotional intimacy and relational satisfaction in couples. The absence of shared bedtime routines when one partner retires earlier has been demonstrated to result in feelings of isolation or disconnection, potentially exacerbating stress and negatively effecting sleep quality.^25^ These dynamics highlight the complex interplay between relational and environmental factors on sleep outcomes within the dyadic context.

### Gender-specific vulnerabilities

A salient contribution of this study is the identification of disproportionate declines in sleep outcomes among women in misaligned bedtime arrangements, providing the first longitudinal evidence of gender-specific vulnerabilities in couple sleep dynamics. This finding addresses a critical gap identified in previous research, where women reported 1.4 times higher rates of insomnia compared with men,^11^ yet the mechanisms underlying this disparity in shared sleep environments remained poorly understood.

The mechanistic basis for women’s greater vulnerability to bedtime misalignment may stem from divergent biological pathways, such as post-menopausal declines in female sex hormones,^12,26^ heightened sensitivity due to increased autonomic nervous system reactivity^25^ and larger circadian misalignments between their central body clock and sleep–wake cycle compared with men.^13^ Additionally, women may be more sensitive to relational and environmental sleep disruptors.^25,27^ For instance, obstructive sleep apnoea, more prevalent in men, often disrupts women’s sleep due to associated nocturnal behaviours.^25^ Conversely, men’s sleep disturbances may be more influenced by their own behaviours rather than by their partner’s bedtime patterns,^28^ which may explain the weaker longitudinal associations observed for men in our study.

These gender-specific vulnerabilities underscore the need for tailored interventions that address the unique sleep challenges faced by women in co-sleeping arrangements. The incorporation of gender-sensitive strategies, such as the promotion of synchronised bedtimes or the mitigation of disruptive stimuli during the vulnerable partial overlap periods, holds promise for more effective improvement of sleep health in older couples.

### Implications for clinical practice and public health

Sleep disturbances represent a significant concern among elderly people, affecting nearly 50% of this demographic and increasing the risk of chronic diseases, cognitive decline and depression.^1–4^ However, current therapeutic frameworks rarely address couple-level dynamics in sleep interventions. We were able to find only one review that recommended integration of bed partners into CBT-I.^8^ Because modification of bedtimes through sleep restriction and limiting wakeful stimuli (such as joining a partner in bed) are core components of CBT, the authors suggest that adjusting bedtimes may benefit both individuals, and our results provide supporting evidence. Specifically, our dose–response findings imply that CBT-I should include specific guidance on avoiding partial bedtime overlap, with complete temporal separation beyond sleep onset latency being preferable. Furthermore, previous studies emphasising the greater sensitivity of women to disruptive stimuli^25,27^ argue for greater focus to be given to the needs of women. The integration of synchronised bedtime routines and the consideration of gender-specific needs has the potential to enhance both individual sleep outcomes and relational satisfaction.

These findings underscore the importance of incorporating couple-level insights into public health strategies. Public health initiatives should prioritise the development of sleep education programmes tailored to older couples, emphasising the importance of bedtime alignment and providing practical strategies for achieving optimal timing patterns. The dissemination of evidence-based recommendations – such as maintaining synchronised bedtimes or ensuring sufficient temporal separation beyond the sleep onset latency of the earlier sleeper – has the potential to offer a scalable and cost-effective solution to mitigate sleep disturbances in ageing populations.

Given the US$94.9 billion annual healthcare price tag attributed to sleep disorders in the USA alone, and the substantial societal impacts of sleep disturbances,^29^ interventions targeting bedtime alignment should be considered a priority in public health policy. The clear dose–response relationship we have identified offers simple, actionable guidance that can be disseminated through primary care settings, digital health platforms and community ageing programmes without the requirement of specialised sleep medicine expertise.

### Strengths and limitations

This study is strengthened by its large, well-characterised cohort from KLOSCAD and rigorous statistical analyses that accounted for key demographic and clinical covariates. The novel, 5-category bedtime alignment classification system and 8-year retrospective trajectory analysis provide unprecedented depth in understanding couple sleep dynamics over time.

However, several limitations should be acknowledged. First, the reliance on self-reported sleep metrics may have introduced recall bias, and the lack of objective assessments for sleep outcomes may be particularly notable. Second, the retrospective design precludes causal inferences about the relationship between bedtime alignment and sleep outcomes. Third, sleep efficiency was calculated without consideration of wake-after-sleep-onset, which may have affected the precision of our sleep outcome measures. Fourth, the sample comprised only Korean older adults, which may limit the generalisability of the findings to younger couples or to those from different cultural contexts. Fifth, this is the first application of bedtime alignment classification to be created by the authors. Additional studies will be needed to demonstrate its merits and generalisability.

### Future research directions

This study identifies several avenues for future research that could advance our understanding of couple sleep dynamics. First, prospective longitudinal studies incorporating objective assessments, such as actigraphy or polysomnography, are needed to confirm the temporal relationships between bedtime alignment and sleep outcomes and establish causality. Second, future research should explore the underlying mechanisms of gender-specific vulnerabilities, including hormonal, behavioural and psychosocial factors, to inform the design of tailored interventions. Third, interventional studies are needed to test whether modification of bedtime alignment leads to improved sleep outcomes and enhanced relationship quality. The efficacy of interventions leveraging digital health technologies, such as smartphone applications and wearable devices, should be explored for monitoring and improving bedtime alignment. These tools have the potential to provide real-time feedback to couples, helping them optimise their sleep timing patterns. Fourth, expanding this research to include younger couples and diverse cultural contexts will enhance the generalisability of the findings. In collectivist cultures, where family cohesion is highly valued, the effects of bedtime misalignment on sleep outcomes might differ from those in individualist cultures, requiring culturally adapted intervention approaches. Finally, integration with existing couple therapy and relationship education programmes could provide a comprehensive approach to improving both sleep and relationship quality, addressing the bidirectional associations between sleep parameters and interpersonal interactions that previous research has documented.

In conclusion, this study highlights bedtime alignment as an impactful determinant of sleep outcomes in older couples. By acknowledging the dyadic nature of co-sleeping and addressing gender-specific vulnerabilities, these findings provide a foundation for developing innovative, holistic approaches to managing sleep disturbances in ageing populations. The identification of partial sleep onset overlap as being particularly disruptive, combined with evidence of progressive sleep deterioration in women over time, demand immediate attention from both clinical practitioners and public health policy-makers. Such efforts have the potential to transform both individual and relational well-being, addressing a critical public health challenge in an ageing society.

## Supporting information

An et al. supplementary material An et al. supplementary material

## Data Availability

The data that support the findings of this study are available from the corresponding author, K.W.K., upon reasonable request.

## Supplementary material

The supplementary material is available online at https://doi.org/10.1192/bjo.2026.10982

## Note

The authors made use of ChatGPT-4 (OpenAI) to assist with the drafting of this article. ChatGPT-4 was accessed from www.chatgpt.com and used without modification from November 2024 to February 2025. The authors subsequently reviewed and edited the content as needed, and take full responsibility for the content of the publication.
