# Supplementary material for: The impact of bedtime alignment on sleep health in older couples: gender-sensitive analysis
Source: BJPsych Open. 2026 Feb 20;12(2):e69. doi: 10.1192/bjo.2026.10982 (PMC12926883; doi:10.1192/bjo.2026.10982)
Supplement: An et al. supplementary material [file S205647242610982Xsup001.docx]

**Supplementary Table 1. Calculations of bedtime alignment**

| Situation | Calculation | Example |
| --- | --- | --- |
| Bedtime of both participants before midnight | Later bedtime – earlier bedtime;  compare with sleep onset latency of earlier sleeper | Bedtime of participant 1: 21:30  Bedtime of participant 2: 22:00  22:00 – 21:30 = 30 minutes;  compare with sleep onset latency of participant 1 |
| Bedtime of one participant before midnight and one participant after midnight | (Later bedtime + 24 hours) - earlier bedtime;  compare with sleep onset latency of earlier sleeper | Bedtime of participant 1: 00:30  Bedtime of participant 2: 23:30  00:30 + 24:00 – 23:30 = 60 minutes;  compare with sleep onset latency of participant 2 |
| Bedtime of both participants after midnight | Later bedtime – earlier bedtime;  compare with sleep onset latency of earlier sleeper | Bedtime of participant 1: 02:00  Bedtime of participant 2: 00:30  02:00 – 00:30 = 90 minutes;  compare with sleep onset latency of participant 2 |

**Supplementary Table 2. Demographic, clinical, and sleep characteristics of the participants**

|  | All | Bedtime group^*^ | | | | | | |
| --- | --- | --- | --- | --- | --- | --- | --- | --- |
|  | (n = 859) | I>>S^a^ (n = 274) | I>S^b^ (n = 48) | I=S^c^ (n = 201) | I<S^d^ (n = 55) | I<<S^e^ (n = 281) | *p*^†^ | post-hoc^†^ |
| Age, year |  |  |  |  |  |  |  |  |
| Index | 74.9 (4.8) | 74.8 (4.9) | 76.7 (5.1) | 74.9 (4.8) | 74.9 (4.3) | 74.6 (4.8) | 0.108 |  |
| Spouse | 73.7 (6.1) | 72.6 (6.4) | 75.7 (5.6) | 73.9 (6.0) | 73.2 (5.9) | 74.3 (5.9) | 0.001 | a < b, e |
| *p*^‡^ | <0.001 | <0.001 | 0.364 | 0.092 | 0.075 | 0.523 |  |  |
| Women |  |  |  |  |  |  |  |  |
| Index | 349 (40.6) | 79 (28.8) | 23 (47.9) | 85 (42.3) | 19 (34.5) | 143 (50.9) | <0.001 | a < b, c, e |
| Spouse | 510 (59.4) | 195 (71.2) | 25 (52.1) | 116 (57.7) | 36 (65.5) | 138 (49.1) | <0.001 | b, c, e < a |
| *p*^‡^ | <0.001 | <0.001 | 0.683 | 0.002 | 0.001 | 0.673 |  |  |
| Men |  |  |  |  |  |  |  |  |
| Index | 510 (59.4) | 195 (71.2) | 25 (52.1) | 116 (57.7) | 36 (65.5) | 138 (49.1) | <0.001 | a < b, c, e |
| Spouse | 349 (40.6) | 79 (28.8) | 23 (47.9) | 85 (42.3) | 19 (34.5) | 143 (50.9) | <0.001 | b, c, e < a |
| *p*^‡^ | <0.001 | <0.001 | 0.683 | 0.002 | 0.001 | 0.673 |  |  |
| Education, year |  |  |  |  |  |  |  |  |
| Index | 10.3 (5.0) | 10.1 (5.0) | 10.5 (5.7) | 10.6 (5.1) | 10.6 (5.1) | 10.0 (4.7) | 0.768 |  |
| Spouse | 10.1 (6.2) | 10.3 (8.1) | 9.7 (6.0) | 10.3 (5.2) | 9.6 (4.5) | 10.0 (4.8) | 0.892 |  |
| *p*^‡^ | 0.587 | 0.795 | 0.543 | 0.562 | 0.253 | 0.901 |  |  |
| Physical activity^§^ |  |  |  |  |  |  |  |  |
| Index | 1417 (2068) | 1512 (2202) | 976 (1147) | 1298 (1514) | 1772 (3259) | 1415 (2096) | 0.282 |  |
| Spouse | 1289 (1792) | 1071 (1478) | 947 (1155) | 1403 (1823) | 1194 (1342) | 1497 (2155) | 0.031 | a < e |
| *p*^‡^ | 0.171 | 0.006 | 0.902 | 0.528 | 0.228 | 0.648 |  |  |
| Current drinking^¶^ |  |  |  |  |  |  |  |  |
| Index | 38 (4.4) | 14 (5.1) | 1 (2.1) | 5 (2.5) | 3 (5.5) | 15 (5.3) | 0.493 |  |
| Spouse | 32 (3.7) | 10 (3.6) | 2 (4.2) | 3 (1.5) | 1 (1.8) | 16 (5.7) | 0.170 |  |
| *p*^‡^ | 0.464 | 0.404 | 0.558 | 0.475 | 0.308 | 0.853 |  |  |
| GDS, point |  |  |  |  |  |  |  |  |
| Index | 8.1 (6.2) | 7.6 (6.1) | 11.5 (7.4) | 8.1 (6.3) | 8.4 (5.5) | 8.0 (6.2) | 0.003 | a, c, e < b |
| Spouse | 8.4 (6.3) | 9.4 (6.6) | 9.2 (6.3) | 7.5 (5.6) | 10.8 (6.8) | 7.6 (6.1) | <0.001 | c, e < a, d |
| *p*^‡^ | 0.302 | 0.001 | 0.099 | 0.318 | 0.040 | 0.416 |  |  |
| CIRS, point |  |  |  |  |  |  |  |  |
| Index | 6.7 (3.3) | 6.8 (3.2) | 8.2 (4.1) | 6.6 (3.1) | 7.0 (3.4) | 6.4 (3.4) | 0.010 | c, e < b |
| Spouse | 5.7 (3.2) | 5.8 (3.5) | 6.8 (3.2) | 5.4 (3.0) | 6.4 (3.3) | 5.5 (2.9) | 0.012 | c < b |
| *p*^‡^ | <0.001 | <0.001 | 0.075 | <0.001 | 0.431 | <0.001 |  |  |
| RBDSQ, points |  |  |  |  |  |  |  |  |
| Index | 1.4 (1.8) | 1.3 (1.7) | 1.5 (1.7) | 1.4 (1.9) | 1.9 (2.4) | 1.3 (1.7) | 0.308 |  |
| Spouse | 1.2 (1.7) | 1.3 (1.7) | 1.3 (2.2) | 1.2 (1.7) | 1.9 (1.8) | 1.1 (1.5) | 0.047 | e < d |
| *p*^‡^ | 0.065 | 0.742 | 0.572 | 0.178 | 0.999 | 0.113 |  |  |
| STOP, points |  |  |  |  |  |  |  |  |
| Index | 1.0 (0.9) | 1.0 (0.9) | 1.1 (0.9) | 1.1 (0.9) | 1.1 (0.9) | 0.9 (0.8) | 0.122 |  |
| Spouse | 0.9 (0.9) | 0.9 (0.9) | 1.0 (0.8) | 0.8 (0.8) | 1.1 (0.9) | 0.8 (0.8) | 0.248 |  |
| *p*^‡^ | 0.031 | 0.377 | 0.905 | 0.025 | 0.672 | 0.463 |  |  |
| Bedtime, HH:MM |  |  |  |  |  |  |  |  |
| Index | 22:19 (01:13) | 21:36 (01:08) | 21:54 (01:02) | 22:18 (01:00) | 22:36 (00:57) | 23:00 (01:07) | <0.001 | a, b, c < e |
| Spouse | 22:21 (01:20) | 23:18 (01:20) | 22:30 (00:57) | 22:18 (01:00) | 21:54 (00:56) | 21:30 (01:05) | <0.001 | b, c, d, e <a |
| *p*^‡^ | 0.620 | <0.001 | 0.004 | 0.999 | <0.001 | <0.001 |  |  |
| PSQI, point |  |  |  |  |  |  |  |  |
| Index | 5.9 (3.3) | 5.1 (2.9) | 7.9 (4.1) | 5.9 (3.4) | 5.5 (2.6) | 6.5 (3.5) | <0.001 | a, c, d, e < b |
| Spouse | 6.0 (3.5) | 6.7 (3.6) | 6.7 (3.7) | 5.6 (3.0) | 8.8 (4.1) | 4.9 (3.1) | <0.001 | c, e < a, b < d |
| *p*^**^ | 0.614 | <0.001 | 0.284 | 0.870 | <0.001 | <0.001 |  |  |
| SOL, min |  |  |  |  |  |  |  |  |
| Index | 23.7 (24.6) | 17.9 (15.4) | 53.4 (46.8) | 22.4 (21.1) | 24.1 (20.3) | 25.2 (25.9) | <0.001 | a, c, d, e < b |
| Spouse | 26.4 (30.0) | 31.1 (36.3) | 27.4 (24.0) | 24.0 (21.0) | 63.2 (49.7) | 16.2 (14.0) | <0.001 | e < a, b, c < d |
| *p*^**^ | 0.112 | <0.001 | 0.001 | 0.464 | <0.001 | <0.001 |  |  |
| SE, % |  |  |  |  |  |  |  |  |
| Index | 94.4 (5.9) | 96.1 (3.3) | 88.0 (10.5) | 95.0 (4.5) | 94.3 (4.7) | 93.5 (7.0) | <0.001 | b<a, c, d, e |
| Spouse | 93.8 (7.2) | 92.2 (8.4) | 93.4 (5.7) | 94.3 (5.7) | 85.6 (12.6) | 96.5 (3.2) | <0.001 | d<a, b, c, e |
| *p*^**^ | 0.130 | <0.001 | 0.004 | 0.288 | <0.001 | <0.001 |  |  |

Mean (standard deviation) for continuous variables and as number (percentage) for categorical variables.

GDS, Geriatric Depression Scale; CIRS, Cumulative Illness Rating Scale; RBDSQ, REM sleep Behavior Disorder Screening Questionnaire; STOP, the STOP questionnaire for obstructive sleep apnea; PSQI, Pittsburgh Sleep Quality Index; SOL, sleep onset latency; SE, sleep efficiency

^*^Couples were classified into five mutually exclusive bedtime alignment groups based on the temporal relationship between partners' bedtimes and sleep onset latencies: 1) “I>>S” group in which the index participant retired to bed earlier than their spouse, with the difference in bedtimes exceeding the index participant’s sleep onset latency; 2) “I>S” group where the index participant retired to bed earlier than their spouse, but the difference in bedtimes was shorter than the index participant’s sleep onset latency; 3) “I=S” group consisted of couples who retired to bed at the same time; 4) “I<S” group where the index participant retired to bed later than their spouse, with the difference in bedtimes shorter than the spouse participant's sleep onset latency; and 5) “I<<S” group where the index participant retired to bed later than their spouse, and the difference in bedtimes exceeded the spouse participant's sleep onset latency.

^†^Comparisons between bedtime groups using analysis of variance and the chi-square test, both with Bonferroni post-hoc comparisons. Analyses for the PSQI score, SOL, and SE were adjusted for gender, GDS score, CIRS score for the index participants and gender, age, physical activity, GDS score, CIRS score and RBDSQ score for the spouse participants.

^‡^Comparisons between index and spouse participants using Student’s t-test and the chi-square test.

^§^Metabolic Equivalent Task*min/week

^¶^≥ 21 standard units per week

^**^Comparisons between index and spouse participants using analysis of variance adjusted for gender, age, CIRS score, and STOP score.
